# Evaluating a New Photopheresis System: A Comparison with Two Established Systems on Cell Yield and Collection Efficiency

**DOI:** 10.3390/diagnostics14202290

**Published:** 2024-10-15

**Authors:** Orkan Kartal, Sandra Laner-Plamberger, Eva Rohde, Cornelia Mrazek, Wanda Lauth, Christoph Grabmer

**Affiliations:** 1 Department for Transfusion Medicine, University Hospital of Salzburg, Paracelsus Medical University Salzburg, Muellner Hauptstraße 48, 5020 Salzburg, Austria; o.kartal@salk.at (O.K.); s.laner-plamberger@salk.at (S.L.-P.); e.rohde@salk.at (E.R.); 2 GMP Laboratory, Paracelsus Medical University Salzburg, Strubergasse 21, 5020 Salzburg, Austria; 3 Department of Laboratory Medicine, University Hospital of Salzburg, Paracelsus Medical University Salzburg, Muellner Hauptstraße 48, 5020 Salzburg, Austria; c.mrazek@salk.at; 4 Team Biostatistics and Big Medical Data, IDA Lab, Paracelsus Medical University Salzburg, 5020 Salzburg, Austria; wanda.lauth@pmu.ac.at; 5 Research and Innovation Management, Paracelsus Medical University Salzburg, 5020 Salzburg, Austria

**Keywords:** photopheresis, mononuclear cells, collection efficiency, graft vs. host disease, flow cytometry

## Abstract

**Background/Objectives**: Extracorporeal photopheresis (ECP) is a well-established and efficacious cell therapy for a range of diseases. The objective of this retrospective study was to compare the new Amicus Blue inline system with the Therakos Cellex inline system and the Spectra Optia offline system in terms of collection efficiency, mononuclear cell (MNC) yield of mononuclear cell products (MCPs), processing time and correlation between MCP cell count and peripheral blood count of patients. Methods: This retrospective study compared 127 procedures utilizing the Spectra Optia offline system, 93 procedures employing the Amicus Blue inline system, and 81 procedures applying the Therakos Cellex inline system. The MNCs were subjected to flow cytometry analysis for CD45 and CD14 expression in order to ascertain the precise composition of the collected lymphocyte and monocyte fractions. Results: The Therakos inline system demonstrated the highest MNC collection efficiency (Therakos: 74.42 ± 1.82; Optia: 65.79 ± 1.48; Amicus: 56.32 ± 2.80; *p* < 0.01). Regarding the content of collected MNCs (×10⁶/kg body weight), the Spectra Optia offline system was superior to the other systems (Optia: 42.69 ± 1.42; Therakos: 31.21 ± 1.66; Amicus: 27.56 ± 1.54; *p* < 0.01). Conclusions: This study represents the first direct comparison of the new Amicus Blue inline system with the two most commonly used ECP systems in the same patient cohort of a single center. The data show that the Amicus Blue inline system collects sufficient MNCs to perform an ECP, but it has a significantly lower CE than the other systems and a significantly lower amount of collected MNCs than the Spectra Optia offline system.

## 1. Introduction

Extracorporeal photopheresis (ECP), originally developed for the treatment of cutaneous T-cell lymphoma, is an established therapeutic option for acute and chronic graft-versus-host disease (GvHD), rejection after heart and lung transplantation, and autoimmune diseases [1,2,3,4]. This growing range of therapeutic indications is due to the non-immunosuppressive mechanism and the favorable safety profile with minimal side effects and no long-term complications.

Currently, there are two types of systems in use for the application of ECP. Firstly, there is the offline system, in which mononuclear cells (MNCs) are collected using a conventional apheresis device. In this system, cells are irradiated using a separate UVA illuminator. The second is the inline system, which combines MNC collection, irradiation and reinfusion in a single device. The advantages of the offline system include flexibility in processing different blood volumes and the ability to use different therapeutic protocols on the same cell separator. By using a sterile connector, offline ECP can also be performed as a closed system, reducing the risk of cell product contamination. However, the risk of mix-ups during reinfusion, the lack of a single needle option and the need for upfront validation of the entire system remain as disadvantages [5,6,7,8].

On the other hand, the inline system has the advantage of a shorter procedure time, single needle access and less hands-on time for the operator. The potential disadvantages of the system include higher disposable costs and exclusive use for ECP [9,10].

Recently, a new inline system has been developed using the Amicus apheresis device (Fresenius Kabi, Lake Zurich, IL, USA) for MNC collection [11]. The Amicus separator is an established device for platelet and MNC collection and has already been used for offline ECP. The Amicus Blue ECP system (Fresenius Kabi) is a functionally closed system. The newly developed Phelix UVA illuminator works in conjunction with the apheresis machine using a single disposable kit. In addition, the system uses a bacterial filter to reduce the risk of bacterial contamination during the injection of 8-methoxypsoralen (8-MOP).

The objective of this retrospective study was to undertake a comparative analysis of the Amicus Blue system with two commonly used systems: the Therakos Cellex (Johnson & Johnson, New Brunswick, NJ, USA) inline system and the Spectra Optia (Terumo BCT, Lakewood, CO, USA) offline system using the Macogenic G2 illuminator (Macopharma, Mouvaux, France) for UVA irradiation. The primary parameters employed for the comparative analysis of the three systems were collection efficiency (CE), mononuclear cell product (MCP) yield, processing time, and the correlation between MCP cell count and peripheral blood count in patients.

## 2. Materials and Methods

### 2.1. Sample Collection and Study Design

This retrospective study compared 127 ECP procedures with the Spectra Optia offline system, 93 ECP procedures with the Amicus Blue inline system and 81 ECP procedures with the Therakos Cellex inline system. A total of 9 patients were included who were treated with all three devices alternately, depending on availability. All participants signed informed consent. The study was approved by the Institutional Ethics Committee (Ethikkommission für das Bundesland Salzburg, approval number: 1022/2022). The patients were diagnosed with chronic GvHD following allogeneic stem cell transplantation, bronchiolitis obliterans syndrome (BOS) after lung transplantation, scleroderma and Crohn’s disease. A complete blood count, including white blood cells (WBCs), hemoglobin, hematocrit, platelets, and percentages of neutrophils, lymphocytes, and monocytes, was performed before starting ECP. Information on ECP procedures was collected, including the volume of blood processed, MNC collection time, Anticoagulant Citrate Dextrose Solution (ACD-A) used, MCP volume, ratio of volume of blood processed to total blood volume, and photoactivation time. ECP treatments were in accordance with established guidelines [1,2,3]. Before each session, a medical assessment was performed to rule out any contraindications to ECP. In addition, vital signs such as blood pressure, pulse rate, temperature and weight were routinely monitored.

### 2.2. Spectra Optia Offline System

All offline procedures were performed using double-needle access. ACD-A was used as anticoagulant at a ratio of 1:12 during ECP. Cell collection was performed using the continuous MNC (cMNC) program of the Spectra Optia apheresis. A total of approximately 0.5-fold the patient’s blood volume was processed, following a recent publication by Mayer et al., in which a reduced processed blood volume (<1-fold BV) showed comparable collection results to the inline system [9]. After collecting the MNCs, the MCP was diluted with normal saline to a volume of 300 mL. A sterile connecting device (TSCD-II, Terumo BCT, Lakewood, CO, USA) was used to prevent bacterial contamination. A standardized amount of 5 mL 8-MOP was injected into the irradiation bag, and cells were photoactivated with an UVA illuminator (Macogenic G2; wavelength 365 nm, 2.0 J/cm^2^). The photoactivated MCP was then reinfused into the patient.

### 2.3. Amicus Blue Inline System

Treatment was performed using Amicus software 4.51 processing 2000 mL of whole blood. Double-needle access was used for all apheresis procedures. After cell collection, the apheresis machine automatically diluted the MCP with 170 mL of normal saline. Anticoagulation consisted of ACD-A at a ratio of 1:12. The Amicus Blue protocol had predefined offsets for cell collection, and a fixed volume of 3.4 mL of 8-MOP was added to the illumination bag. The Phelix UVA illuminator (software 1.0) delivered 1.5 J/cm^2^ of UVA light to the MCP, and after photoactivation, treated cells were automatically reinfused into the patient.

### 2.4. Therakos Cellex Inline System

Inline ECP was conducted in accordance with the Therakos CellEx operator’s manual. Treatment was performed using software 5.4 processing 1500 mL of whole blood at a flow rate of 30 to 50 mL/min using a whole blood/ACD-A ratio of 12:1. The ECP system utilizes disposable MNC collection sets that use either a single or double needle for blood collection and a Latham bowl for cell enrichment. After the collection of MNCs, the 8-MOP to be injected was calculated automatically by the software. After the illumination process, the MNCs were reinfused into the patient.

### 2.5. Determination of Cell Blood Counts and CE

A Sysmex XN-9000 hematology analyzer (Sysmex Corporation, Kobe, Japan) was used to determine the complete cell blood count, including neutrophils, lymphocytes, and monocytes in the venous blood, as well as the WBC count of the MCP for each system. The CE was assessed using the CE 2 method (%), which was calculated as follows for each system: (WBCs [or lymphocytes, or monocytes, or MNCs, or neutrophils] collected [×10^6^/mL] × product volume [mL])/(pre-apheresis WBCs [or lymphocytes, or monocytes, or MNCs, or neutrophils] [×10^6^/mL] × [processed volume − volume ACD-A]) × 100.

### 2.6. Flow Cytometry

To determine the absolute number of lymphocytes and monocytes in the MCP, CD45 and CD14 expression was determined by flow cytometry as previously described [12].

### 2.7. Statistical Analysis

For the descriptive analysis, mean values and standard errors were calculated and compared. To determine the strength of the relation between the peripheral and MCP values, the Pearson correlation coefficient was calculated. An absolute value of the correlation of less than 0.2 was defined as very weak (to none), between 0.2 and 0.4 as weak, between 0.4 and 0.6 as moderate, between 0.6 and 0.8 as strong and greater than 0.8 as very strong. To illustrate this linear dependency more clearly, linear regressions were performed, and the coefficient of determination (R-squared) as well as the intercept and slope were compared for each system. For this purpose, the three systems were stratified. The differences between the systems in terms of CE and MCP values were statistically determined using the non-parametric Kruskal–Wallis test. If the results were significant, the Dunn test [13] was used as a post hoc test. To account for multiplicity, the Bonferroni–Holm correction was employed to adjust for multiple testing. The two-sided significance level α = 0.05 was applied to all hypothesis tests with the significance levels indicated in the plots as follows: *p* < 0.05: *, *p* < 0.01: **, *p* < 0.001: ***. All calculations were carried out using the statistical software R (Version 4.3.2) [14].

## 3. Results

### 3.1. Patient Data and MNC Collection

During the study period from September 2021 to February 2024, a total of 162 ECP procedures were performed in nine patients. This retrospective study focused on the direct comparison of three different ECP systems: 127 ECP procedures were performed with the Spectra Optia offline system (Optia), 93 ECP procedures with the Amicus Blue inline system (Amicus) and 81 ECP procedures with the Therakos Cellex inline system (Therakos) (Table 1). All ECP procedures were well tolerated by the patients with no serious side effects.

Table 2 shows the pre-procedural peripheral blood cell values and the ECP procedure data. There was no statistically significant difference in pre-procedural peripheral blood cell counts between the three systems studied (*p* > 0.05). This means that the patients were comparable in terms of their blood cell counts before apheresis, which indicates that there is no bias due to differences in the initial blood count of the patients and that the results of subsequent apheresis can therefore be evaluated under the same initial conditions.

In contrast, the ECP procedure data showed statistical significance in almost all parameters evaluated (*p* < 0.01), with the exception of procedure runtime between the Amicus and Therakos, where no significant difference was found. This suggests that the systems perform differently in most aspects of the procedure. These differences may be due to the specific technical characteristics of each independent system.

However, it should be noted that the runtime listed for the Optia only reflects the MNC collection time without considering irradiation and reinfusion. As reported in the study by Mayer et al. [9], about 45 min should be added to the offline procedure runtime to include the additional irradiation and handling steps.

### 3.2. MCP Characteristics and Collection Efficiency

The analysis of MNCs × 10^6^/kg body weight (BW) shows the highest values when using the Optia (42.69 ± 1.42), followed by Therakos (31.21 ± 1.66) and Amicus (27.56 ± 1.54), with the differences between Optia and Therakos and between Optia and Amicus being statistically significant (*p* < 0.01). In contrast, no statistically significant difference was found between Amicus and Therakos (*p* > 0.05). The data indicate that the Optia is likely to collect the highest number of MNCs in a similar amount of time (Table 3).

A similar pattern is seen for lymphocytes and monocytes × 10^6^/kg BW, with the highest values being observed for Optia, followed by Therakos and Amicus. In these cases, the differences between Optia and the other two systems are also statistically significant (*p* < 0.01), whereas no significant differences are observed between Amicus and Therakos (*p* > 0.05). 

Additionally, all other values show statistically significant differences (*p* < 0.05) between Optia and the other two systems (Table 3). However, while the lymphocytes, neutrophils, WBCs, monocytes, and MNCs × 10^9^/L in the MCP are statistically significantly different (*p* < 0.01) between Amicus and Therakos, their values per kilogram of BW are not statistically significant (*p* > 0.05).

**Figure 1:**
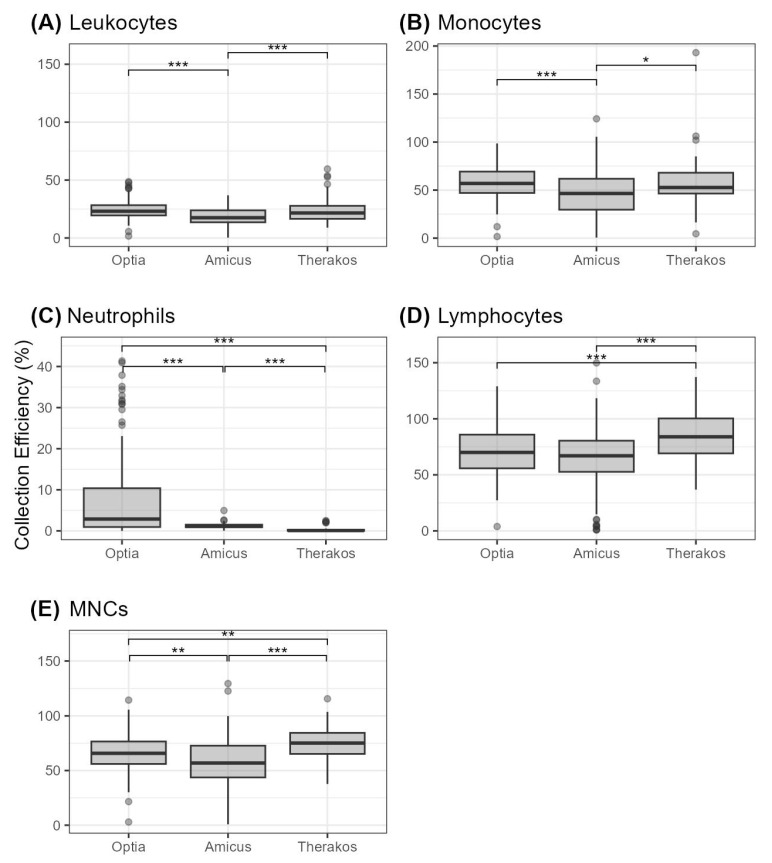
Cell collection efficiency (given in percent) for different cell types depicted as boxplots (**A**) leukocytes, (**B**) monocytes, (**C**) neutrophils, (**D**) lymphocytes, and (**E**) MNCs. Statistical significance is indicated by * (*p* < 0.05), ** (*p* < 0.01) and *** (*p* < 0.001).

Our data further reveal that the collection efficiency (CE) for MNCs are highest for Therakos with 74.42 ± 1.82%, which is followed by Optia with 65.79 ± 1.48% and Amicus with 56.32 ± 2.80% (Table 4). These differences are statistically significant, as shown in Figure 1.

Therakos also has the highest CE for lymphocytes (85.07 ± 2.46%), while Optia follows with 70.98 ± 1.97% and Amicus with 63.39 ± 3.11%. It should be noted that there are no statistically significant differences between Optia and Amicus. Regarding neutrophils, monocytes and WBCs, the Optia demonstrated the highest CE with values of 7.96 ± 1.97% for neutrophils, 56.36 ± 1.44% for monocytes and 24.81 ± 0.78% for WBCs. These results are statistically significant compared to the Amicus (Figure 1). Therakos shows the second highest values for the collection of these cell types, but the CE values for leukocytes and monocytes are not statistically significantly different compared to the Optia.

### 3.3. Correlation and Linear Regression between Peripheral and MCP Cell Counts

**Figure 2:**
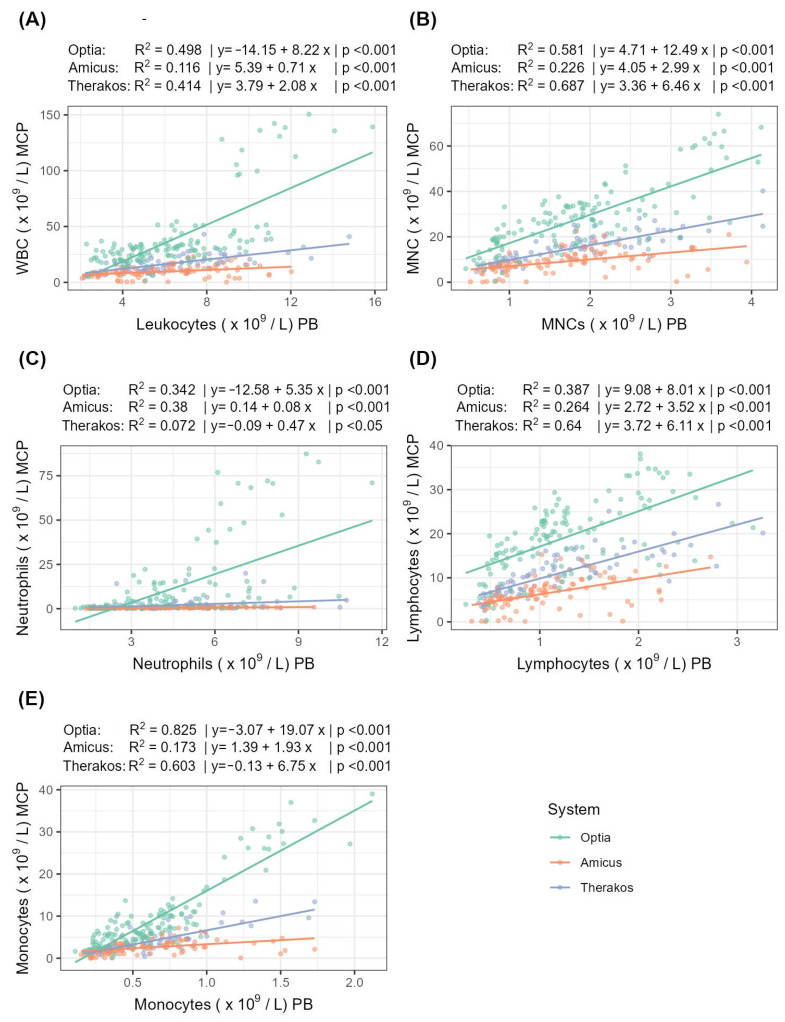
Correlation and linear regression between peripheral and MCP cell counts for (**A**) WBCs, (**B**) MNCs, (**C**) neutrophils, (**D**) lymphocytes, and (**E**) monocytes. R^2^ indicates the strength of the linear regression.

The following section examines the correlation and linear regression of the peripheral and MCP cell counts for the three systems (Table 5, Figure 2).

For MNCs, Therakos shows a very strong correlation strength, while Optia shows a strong correlation and Amicus shows only a moderate correlation. A similar pattern can be seen for lymphocytes and monocytes: for lymphocytes, the correlation strength is strong for both Optia and Therakos. For monocytes, the correlation for Therakos is strong, while for Optia, it is very strong. In contrast, the correlation for neutrophils is quite different. Here, Amicus achieves a strong correlation, while Optia shows only a moderate correlation and Therakos shows a weak one. The analysis of the WBCs correlation strength reveals that the Optia and Therakos systems strongly correlate, while the Amicus system only achieves a weak correlation. 

## 4. Discussion

The range of indications for ECP has expanded significantly in recent decades, since it allows for an effective treatment of various diseases with an excellent safety profile. To our knowledge, this is the first published study, which compares the new Amicus Blue ECP system with the Therakos Cellex inline system and the Spectra Optia offline system in the same patient cohort of a single center. Like the results previously published by Radwanski et al., we found the Amicus system to be safe, and the procedure was well tolerated with no serious side effects [11].

The disposable set of Amicus has a very low extracorporeal volume of about 163 mL in comparison to the Spectra Optia (253–297 mL) and the Therakos Cellex (255–415 mL in double-needle mode) [11,15,16]. This circumstance makes the system particularly suitable for patients with very low body weight.

In the present study, the Amicus Blue system showed a median CE for MNCs of 56.32%. This confirms the previously published data from Radwanski et al. [11]. However, we observed significantly higher values for the CE for both the Spectra Optia system and the Therakos Cellex system (Optia: 65.79%; Therakos: 74.42%). This is in contrast to the previously published CE data for the Spectra Optia reported by Cid et al. with 42% [17]. Similar values for the CE of around 60% for both systems were reported by Brosig et al. and Piccirillo et al. [5,10]. Differences in CE could be due to the fact that the collected MNCs are usually determined by means of conventional blood cell counting. These hematology analyzers are often not validated to accurately measure apheresis products with high MNC content. In our study, we used flow cytometry for the determination, which is the gold standard for analyzing the expression of cell surface molecules and determining various cell types in a heterogeneous apheresis product [12]. In addition, the CE depends largely on whether the MNC or cMNC protocol of Spectra Optia was used to collect the cells [7].

The high CE of the Therakos system is probably explained by the fact that this device works with a different apheresis technology in which cell enrichment is carried out in a Latham bowl from a very small volume of 1500 mL. The disadvantage of this technology, however, is that the extracorporeal volume is higher than with the other systems. In addition, the patient must have a minimum hematocrit of 27%.

The differences in the CE of the individual systems are also reflected by the correlations between the peripheral blood cell count and the MCP. The Amicus Blue system showed only a moderate correlation between the peripheral MNC count and the collected MNC in the MCP. In contrast, the Spectra Optia as well as the Therakos system showed strong to very strong correlations. Similar results were also observed for the WBC, lymphocyte and monocyte fractions.

With regard to the MNC content per kg BW of the apheresis products, our study showed the highest values for the Spectra Optia system. The measured values were significantly higher compared to those of both the Therakos system and the Amicus system. In any case, all MNC yields were above the recommended thresholds (13.9 × 10^6^ MNCs per kg BW) published by Worel et al. in 2018 [18].

In this study, only 0.5 times the total body volume was processed with the Spectra Optia system. Despite the significant reduction in the volume of blood processed, the highest number of MNCs was collected using the offline system. This supports the recently revised American Society for Apheresis guidelines, which state that a single volume of blood processed with the offline system is equivalent to a cycle of two treatments with an inline system [1].

An important aspect for both physicians and patients is the duration of each individual treatment session. The Therakos system and the Amicus system both have very short treatment times of about 90 min. By reducing the processed blood volume in the offline system, shorter apheresis times can also be achieved, and the total treatment duration is only slightly longer than that of the inline systems. If the therapy cycle is also reduced from two to one treatments per cycle in the offline system, the burden on the patient can be significantly reduced even further. There is also no need for the patient to be hospitalized, which leads to an improvement for the patient and to cost savings.

Apart from costs for hospitalization, the published data show that inline systems cost almost twice as much per treatment as offline systems. If the number of treatments is reduced from two to one per cycle when using the offline system, this saving potential is even greater. Of course, this also depends on the respective Good Manufacturing Practice (GMP) regulations of the individual countries and whether the apheresis products of the offline system are declared as Advanced Therapy Medicinal Products (ATMPs) by the authorities and therefore have to fulfill higher requirements [19,20,21].

## 5. Conclusions

This is the first direct comparison of the new Amicus Blue system with the two most commonly used ECP systems, the Spectra Optia offline system and Therakos Cellex inline system. The new ECP system proved to be safe and was well tolerated by the patients. The CE and the amount of MNCs collected were above the published thresholds but below those of the other two systems. The main advantages over the Therakos system are a lower extracorporeal volume and the possibility of widespread use as an apheresis device for other applications. The possibility of using the single needle method in the latest Amicus Blue software version 6.1 distinguishes it from the Spectra Optia system. In view of the treatment costs, the Spectra Optia system is probably preferable to the other two systems.

## Figures and Tables

**Table 1:** Patient characteristics.

Patients	
*n*	9
Age in years (mean ± SEM)	50.83 ± 0.91
Sex M/F (*n*)	4/5
**Patients disease (*n*)**	
GvHD	3
BOS	3
Crohn’s disease	2
Systemic sclerosis	1
Weight in kg (mean ± SEM)	65.35 ± 0.81
Total blood volume in mL (mean ± SEM)	4119.67 ± 297.46

**Table 2:** Pre-procedure peripheral blood cell count and procedure data (mean ± SEM).

Pre-Procedure Peripheral Blood Cell Count (Mean ± SEM)	Optia	Amicus	Therakos
WBCs (×10^9^/L)	6.53 ± 0.24	5.96 ± 0.25	6.26 ± 0.28
Hb (g/dL)	12.51 ± 0.10	12.23 ± 0.15	11.93 ± 0.15
Hct (%)	37.41 ± 0.23	36.86 ± 0.40	36.44 ± 0.42
Plts (×10^9^/L)	273.69 ± 8.79	255.86 ± 11.98	270.09 ± 11.58
Neutrophils (%)	67.21 ± 1.05	67.96 ± 1.13	67.68 ± 1.37
Lymphocytes (%)	20.23 ± 0.86	19.49 ± 0.88	19.88 ± 1.15
Monocytes (%)	9.85 ± 0.31	10.24 ± 0.37	9.82 ± 0.41
MNC (%)	30.08 ± 1.02	29.73 ± 1.09	29.71 ± 1.37
**ECP (mean ± SEM)**			
*n*	127	93	81
Processed blood volume (mL)	2349.62 ± 42.53	2016.56 ± 8.75	1542.10 ± 2.25
Procedure runtime (minutes)	63.47 ± 0.60 *	92.86 ± 1.05	93.79 ± 2.22
ACD-A used (mL)	202.94 ± 3.79	167.85 ± 1.42	187.74 ± 0.51
MCP volume (mL)	99.82 ± 0.20	200 ± 0.00	132.91 ± 1.71
Processed blood volume/TBV	0.56 ± 0.01	0.48 ± 0.01	0.38 ± 0.01
Photoactivation time (minutes)	9.41 ± 0.05	22.23 ± 0.30	19.07 ± 1.08

* MNC collection time without irradiation and reinfusion.

**Table 3:** MCP concentrate characteristics (mean ± SEM) as determined by flow cytometry.

	Optia	Amicus	Therakos
*n*	127	93	81
MCP volume, mL	99.82 ± 0.20	200 ± 0.00	132.91 ± 1.71
WBCs (×10^9^/L)	39.51 ± 2.81	9.63 ± 0.52	16.81 ± 0.89
WBCs (×10^6^/kg BW)	57.92 ± 2.82	29.09 ± 1.58	35.75 ± 2.21
WBCs (×10^9^)	3.94 ± 0.28	1.93 ± 0.10	2.22 ± 0.12
MNCs (×10^9^)	2.82 ± 0.13	1.83 ± 0.10	1.95 ± 0.09
MNCs (×10^6^/kg BW)	42.69 ± 1.42	27.56 ± 1.54	31.21 ± 1.66
MNCs (%)	79.81 ± 1.97	90.43 ± 1.63	90.70 ± 1.43
Neutrophils (%)	20.27 ± 1.97	9.55 ± 1.63	9.3 ± 1.43
Lymphocytes (×10^6^/kg BW)	29.49 ± 0.96	19.89 ± 1.12	23.26 ± 1.35
Lymphocytes (%)	56.98 ± 1.78	65.54 ± 1.39	67.83 ± 1.75
Monocytes (×10^6^/kg BW)	13.34 ± 0.76	7.67 ± 0.49	7.95 ± 0.58
Monocytes (%)	22.83 ± 0.75	24.89 ± 0.77	22.88 ± 0.96

**Table 4:** Assessment of collection efficiency (given as mean percentage values ± SEM).

	Optia	Amicus	Therakos
WBCs	24.81 ± 0.78	17.46 ± 0.89	24.03 ± 1.13
MNCs	65.79 ± 1.48	56.32 ± 2.80	74.42 ± 1.82
Lymphocytes	70.98 ± 1.97	63.39 ± 3.11	85.07 ± 2.46
Monocytes	56.36 ± 1.44	45.46 ± 2.60	55.89 ± 2.66
Neutrophils	7.96 ± 1.97	1.26 ± 0.06	0.27 ± 0.06

**Table 5:** Correlation and linear regression of peripheral and MCP cell count.

Cell Type	System	Correlation Coefficient, r	Correlation Strength	Linear Regression, R^2^	Slope
WBCs	Optia	0.71	strong	0.498	8.22
	Amicus	0.34	weak	0.116	0.71
	Therakos	0.64	strong	0.414	2.08
MNCs	Optia	0.76	strong	0.581	12.49
	Amicus	0.48	moderate	0.226	2.99
	Therakos	0.83	very strong	0.687	6.46
Neutrophils	Optia	0.58	moderate	0.342	5.35
	Amicus	0.62	strong	0.38	0.08
	Therakos	0.27	weak	0.072	0.47
Lymphocytes	Optia	0.62	strong	0.387	8.01
	Amicus	0.51	moderate	0.264	3.52
	Therakos	0.8	strong	0.64	6.11
Monocytes	Optia	0.91	very strong	0.825	19.07
	Amicus	0.42	moderate	0.173	1.93
	Therakos	0.78	strong	0.603	6.75

## Data Availability

The data presented are not publicly available to protect the privacy of patients.
